# Inhibition of LRRK2 or Casein Kinase 1 Results in LRRK2 Protein Destabilization

**DOI:** 10.1007/s12035-018-1449-2

**Published:** 2018-12-27

**Authors:** T. De Wit, V. Baekelandt, E. Lobbestael

**Affiliations:** Laboratory for Neurobiology and Gene Therapy, Department of Neurosciences, KU Leuven, Herestraat 49 – Bus 1023, 3000 Leuven, Belgium

**Keywords:** Parkinson’s disease, LRRK2, LRRK2 kinase inhibitor, Phosphorylation, Protein stability, Casein kinase 1

## Abstract

Mutations and variations in the leucine-rich repeat kinase 2 (LRRK2) gene are strongly associated with an increased risk to develop Parkinson’s disease (PD). Most pathogenic LRRK2 mutations display increased kinase activity, which is believed to underlie LRRK2-mediated toxicity. Therefore, major efforts have been invested in the development of potent and selective LRRK2 kinase inhibitors. Several of these compounds have proven beneficial in cells and in vivo, even in a LRRK2 wild-type background. Therefore, LRRK2 kinase inhibition holds great promise as disease-modifying PD therapy, and is currently tested in preclinical and early clinical studies. One of the safety concerns is the development of lung pathology in mice and non-human primates, which is most likely related to the strongly reduced LRRK2 protein levels after LRRK2 kinase inhibition. In this study, we aimed to better understand the molecular consequences of chronic LRRK2 kinase inhibition, which may be pivotal in the further development of a LRRK2 kinase inhibitor-based PD therapy. We found that LRRK2 protein levels are not restored during long-term LRRK2 kinase inhibition, but are recovered upon inhibitor withdrawal. Interestingly, LRRK2 kinase inhibitor-induced destabilization does not occur in all pathogenic LRRK2 variants and the N-terminal part of LRRK2 appears to play a crucial role in this process. In addition, we identified CK1, an upstream kinase of LRRK2, as a regulator of LRRK2 protein stability in cell culture and in vivo. We propose that pharmacological LRRK2 kinase inhibition triggers a cascade that results in reduced CK1-mediated phosphorylation of yet unidentified LRRK2 phosphorylation sites. This process involves the N-terminus of LRRK2 and ultimately leads to LRRK2 protein degradation.

**Electronic supplementary material:**

The online version of this article (10.1007/s12035-018-1449-2) contains supplementary material, which is available to authorized users.

## Background

Leucine-rich repeat kinase 2 (LRRK2) is a very attractive target in the development of disease-modifying strategies for Parkinson’s disease (PD). Pathogenic mutations in the LRRK2 gene are the most common cause of inherited forms of PD, and genetic variations in the LRRK2 locus contribute to the risk of developing sporadic forms of the disease [1–9]. All pathogenic mutations (N1437H, R1441C/G/H/S, Y1699C, G2019S, and I2020T) [10, 11] cluster in the catalytic core of the protein that consists of the GTPase domain ROC (Ras of complex proteins), which is linked via the COR domain (C-terminal of ROC) to a kinase domain [12]. This suggests that altered LRRK2 activity is involved in PD pathogenesis, which is supported by the fact that most pathogenic mutations (N1437H, R1441C/G/H, Y1699C, G2019S, and I2020T) display increased LRRK2 kinase activity in cells and in vivo [13–16]. LRRK2-related toxicity in cell culture [17, 18] and in rodent models [19, 20] has been related to augmented LRRK2 kinase activity and can be rescued by LRRK2 kinase inhibitors [13, 19–22]. Interestingly, LRRK2 kinase inhibition is also believed to be beneficial in PD cases without genetic LRRK2 modifications given the increased autophosphorylation at S1292 observed in urinary exosomes [23, 24] and substantia nigra dopaminergic neurons in postmortem brain tissue from patients with idiopathic PD [25]. Moreover, LRRK2 kinase inhibition was shown to protect against α-synuclein-induced toxicity in rodent brain [20]. Consequently, specific and potent LRRK2 kinase inhibitors [26, 27] are considered one of the prevalent disease-modifying therapeutic agents for PD and are currently tested in preclinical studies and phase I clinical trials.

Of particular interest are the molecular consequences of pharmacological LRRK2 kinase inhibition. We and others have shown that LRRK2 becomes ubiquitinated [28] and directed for proteasomal degradation upon LRRK2 kinase inhibition [29], resulting in reduced LRRK2 protein levels [28–33]. How pharmacological inhibition can induce LRRK2 protein degradation is not completely understood. LRRK2 kinase activity has been proposed as a regulator of LRRK2 stability, which is supported by the finding that transgenic knock-in mice expressing a kinase-dead form display decreased LRRK2 protein levels [30]. This is consistent with the recent observations that functional mutants L728D and L729D in the ankyrin domain of LRRK2 show both decreased kinase activity and decreased LRRK2 protein stability [34]. Moreover, preclinical safety studies revealed that LRRK2 kinase inhibitors induce morphological changes in the lungs of mice [32] and non-human primates [33] that resemble the lung phenotype in LRRK2 knock-out mice [35]. However, destabilization upon LRRK2 kinase inhibition is not observed in all conditions [20, 28, 29, 32, 33] and not all animals with affected lungs display decreased LRRK2 levels [33], which indicates that inhibition of LRRK2 kinase activity alone is not sufficient to initiate protein destabilization.

Dephosphorylation at S935 has been suggested to regulate LRRK2 destabilization [28], given that dephosphorylation of heterologous phosphorylation sites, mediated by protein phosphatase 1 (PP1) [36], is a well-validated consequence of LRRK2 kinase inhibition [37–41]. However, we observed that mutating S935 to an alanine residue does not affect inhibitor-induced destabilization [29], which points to a more complex, yet unidentified, relation between LRRK2 S935 dephosphorylation and LRRK2 protein degradation.

Understanding how LRRK2 protein degradation is regulated may be crucial for understanding potential side effects of LRRK2 kinase inhibitors and at the same time reveal new therapeutic clues. In the present study, we aimed to gain better insight in the mechanism of LRRK2 kinase inhibitor-induced destabilization. We show that LRRK2 protein levels are not restored during sustained inhibition, but that this effect is reversible after inhibitor withdrawal. We provide strong evidence that dephosphorylation of the well-characterized heterologous phosphorylation sites is not crucial for inhibition-induced LRRK2 protein destabilization, and that the N-terminus of LRRK2 is presumably involved. We identified casein kinase 1 (CK1) as a regulator of LRRK2 protein stability in cell culture and in vivo. In addition, we show that several pathogenic mutants do not destabilize upon inhibition of LRRK2 kinase activity, but retain sensitivity to CK1 inhibition. Together, our results suggest that LRRK2 homeostasis in cell culture and in vivo relies on a complex, multifactorial mechanism that involves phosphorylation of yet unidentified phosphorylation sites regulated by CK1.

## Materials and Methods

### Antibodies, Plasmids, and Reagents

LRRK2 kinase inhibitors CZC-25146 and PF-06447475 and casein kinase 1 inhibitor IC261 were purchased from Sigma-Aldrich. Casein kinase 1 inhibitor PF-670462 was purchased from Abcam. LRRK2 kinase inhibitor MLi-2 was kindly provided by Dr. D. Alessi (Division of Signal Transduction Therapy, University of Dundee). pCHMWS_3Flag_LRRK2_Ires_Hygro constructs of pathogenic (R1441C/G, Y1699C, and I2020T) LRRK2 variants were cloned as described in [42]. The pCHMWS_3Flag_LRRK2_Ires_Hygro constructs encoding truncated variants PLRCKW and APLRCKW, as well as the LRRK2 S908A/S910A/S935A/S955A/S973A/S976A or S908E/S910E/S935E/S955E/S973E/S976E were generated using gBlock® Gene Fragments (IDT) and as described in [42]. The following antibodies were used: mouse anti-FlagM2 (Sigma-Aldrich, F1804), mouse anti-vinculin (Sigma-Aldrich, V9131), mouse anti-α-tubulin (Sigma-Aldrich, T5168), rabbit anti-LRRK2 P-S935 (Abcam, ab133450), rabbit anti-LRRK2 P-S1292 (Abcam, ab203181), rabbit anti-LRRK2 MJFF-2 antibody (Abcam, ab133474), mouse anti-LRRK2/Dardarin, N-terminus N138/6 (Neuromab 75-188), mouse anti-LRRK2/Dardarin, C-terminus N241A/34 (Neuromab 75-253), mouse anti-LRRK2 MC.028.83.76.242 (ab130277).

### Viral Vector Production

Lentiviral (LV) vectors encoding human 3Flag-LRRK2 variants under control of the cytomegalovirus (CMV) promoter were produced as described previously [43] by our in-house Leuven viral vector core (https://gbiomed.kuleuven.be/english/research/50000715/laboratory-of-molecular-virology-and-gene-therapy/lvvc).

### Cell Culture

SH-SY5Y cells were maintained in Dulbecco’s modified Eagle’s medium (DMEM) (Gibco-life technologies), supplemented with 15% fetal calf serum (Gibco), 1× non-essential amino acids (Gibco), and 50 μg/mL gentamycin at 37 °C in a humidified atmosphere containing 5% CO_2_. All cultures were mycoplasma-free. To generate SH-SY5Y cells stably overexpressing 3Flag-LRRK2 variants, we transduced SH-SY5Y cells with LV vectors and selected them in DMEM supplemented with 200 μg/mL hygromycin. For compound treatment, cells were treated in a 24-well plate for the indicated period of time with the compound indicated or DMSO as negative control. To obtain LRRK2 kinase inhibition, cells were treated with either 200 nM CZC-25146, 150 nM PF-06447475, or 10 nM MLi-2. To obtain inhibition of casein kinase 1, cells were treated with 300 μM IC261 [44]. For washout experiments, cells were rinsed twice with PBS before they were given fresh medium without compound. For cell lysis, cells were rinsed with PBS and lysed on ice in lysis buffer composed of Tris 20 mM pH 7,5, 400 mM NaCl, 1 mM EDTA, 1% Triton, 10% glycerol, protease inhibitor cocktail (Roche), and phosphatase inhibitors (PhosStop, Roche). Cell lysates were cleared by centrifugation at 14000*g* for 10 min and further analyzed via immunoblotting.

### Tissue Extraction

All animal experiments were performed in accordance with the European Communities Council Directive of November 24, 1986 (86/609/EEC) and approved by the Bioethical Committee of the KU Leuven (Belgium). Whole brain, lung, and kidney extracts of C57Bl/6J mice (WT or LRRK2^−/−^) were lysed in sucrose buffer (10 mM Tris-HCl, 1 mM EDTA, 0,25 mM sucrose, protease inhibitor cocktail, and phosphatase inhibitor) using a Dounce homogenizer. Extracts were cleared by 10 min centrifugation at 3000*g*, followed by centrifugation of the supernatant for 30 min at 20000*g*. For experiments using LRRK2 kinase inhibition or CK1 inhibition, C57Bl/6J WT mice were injected i.p. with 10 mg/kg MLi-2, 50 mg/kg PF-670462, or DMSO in 20% hydroxypropyl-β-cyclodextrin (Sigma-Aldrich) and PBS. Four injections were given over a time interval of 30 h and animals were sacrificed 2 h after the last injection.

### Immunoblotting

Protein concentration of cell lysates was determined using the bicinchoninic acid (BCA) protein determination assay (Pierce Biotechnology). Cell lysates were resolved by electrophoresis on 3–8% Criterion™ XT Tris-Acetate protein gels (Bio-Rad). Separated proteins were transferred to a polyvinylidene fluoride membrane (Bio-Rad) and non-specific binding sites were blocked for 15 min in PBS with 0.1% Triton X-100 (PBS-T) and 5% non-fat milk. After overnight incubation at 4 °C with primary antibodies, blots were washed 3 times with PBS-T and incubated with horseradish peroxidase-conjugated secondary antibody (Dako, Glostrup) for 1 h and washed again 3 times. Bands were visualized using enhanced chemiluminescence (Amersham Pharmacia Biotech). To normalize the signal of phospho-specific antibodies to LRRK2 expression levels, blots were stripped after detection of the LRRK2 signal and reprobed with anti-phospho-LRRK2 antibody by incubating the blot with stripping buffer (62.5 mM Tris-HCl pH 6.8, 2% SDS, and 100 mM β-mercaptoethanol) for 30 min at 70 °C, followed by 2 × 10 min wash steps with PBS-T.

### Statistics

Blots shown are representative of at least three independent experiments. LRRK2 phosphorylation levels were normalized to LRRK2 expression levels, LRRK2 protein levels to housekeeping proteins and experimental test conditions to control conditions. Statistical analysis was performed with a 2-way ANOVA test with Bonferroni post-test or column statistics (one-sample *t* test) comparing test values to the hypothetical value of 1. If different treatment terms were applied, significance is only shown for the 48-h time point. Statistical significance: *** *p* < 0.001, ** *p* < 0.01, *p < 0.05.

## Results

### LRRK2 S935 Phosphorylation and Protein Levels Do Not Recover during Chronic Pharmacological LRRK2 Kinase Inhibition

**Fig. 1:**
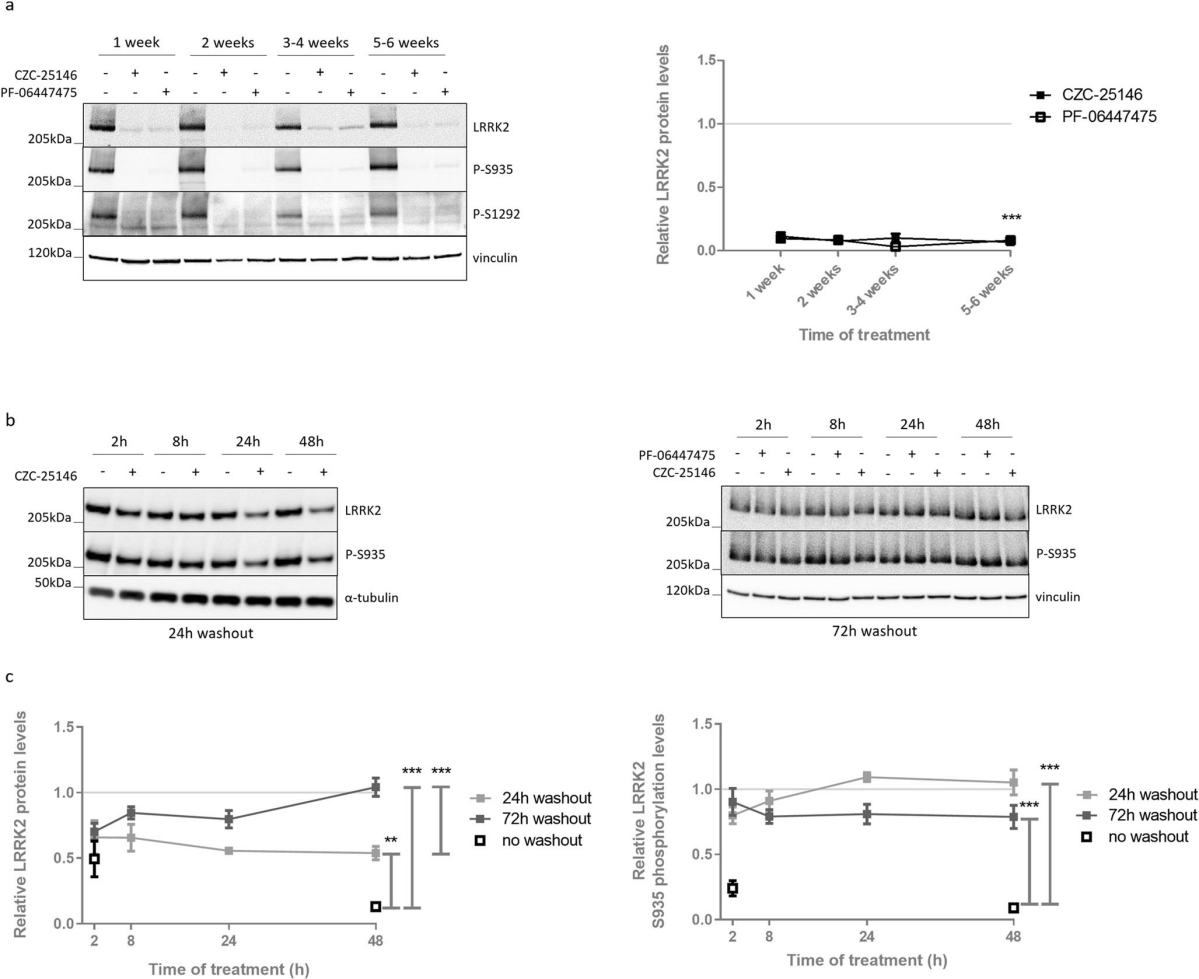
Long-term LRRK2 kinase inhibitor treatment induces a sustained reduction in LRRK2 protein and phosphorylation levels, which can be completely restored after inhibitor removal. SH-SY5Y cells overexpressing 3Flag-LRRK2 WT were treated for indicated terms with CZC-25146 (200 nM), PF-06447475 (150 nM), or DMSO as control. Cell lysates were taken (**a**) on different days during several weeks of treatment or (**b**) 24 h or 72 h after removal of the LRRK2 kinase inhibitor and analyzed with immunoblotting using the FlagM2 antibody for LRRK2 detection, anti-LRRK2 P-S935, anti-LRRK2 P-S1292, anti-α-tubulin, or anti-vinculin for checking equal loading. Representative blots are shown. (**a**, **c**) Graphs show the quantification of blots representing the ratio of total LRRK2 over housekeeping proteins or the ratio of phosphorylation at S935 over total LRRK2 signal. Error bars indicate S.E.M. with *N* ≥ 3. Statistical significance was tested using (**a**) a 2-way ANOVA test with Bonferroni post-tests or (**c**) a Mann-Whitney *U* test. Triple asterisks indicate *p* < 0.001, double asterisks indicate *p* < 0.01

As LRRK2 kinase inhibitor treatment is considered one of the prevailing disease-modifying strategies for PD, insight in the consequences of chronic treatment will be crucial. We have previously shown that LRRK2 kinase inhibition induces degradation of LRRK2 protein starting after 8 h until at least 48 h of treatment [29]. To investigate whether LRRK2 protein levels and S935 phosphorylation remain low during sustained LRRK2 kinase inhibition, we treated SH-SY5Y cells overexpressing LRRK2 WT with the LRRK2 kinase inhibitors CZC-25146 (200 nM) or PF-06447475 (150 nM) over a period of 6 weeks. LRRK2 kinase inhibition was confirmed by dephosphorylation at S935 and S1292. No compensatory effects were observed as both phosphorylation levels at S935, S1292, and total LRRK2 protein levels remained strongly decreased (Fig. 1a).

To test whether the observed effects of LRRK2 kinase inhibition are reversible, cells were treated for different time periods with CZC-25146 or PF-06447475, followed by a washout period of 24 h or 72 h (Fig. 1b, c). Phosphorylation at S935 was completely restored during the washout period. Compared to inhibitor treatment without washout, incomplete recovery of LRRK2 protein levels was observed 24 h after removal of the inhibitor, while LRRK2 levels were fully restored after 3 days of inhibitor withdrawal. This is in line with the relatively long half-life reported for LRRK2 [28, 45–47] as the reduction in LRRK2 protein levels is caused by proteasomal degradation [29] and hence, recovery relies on de novo protein synthesis.

### The N-Terminus of LRRK2 Is Involved in Inhibitor-Induced LRRK2 Destabilization

**Fig. 2:**
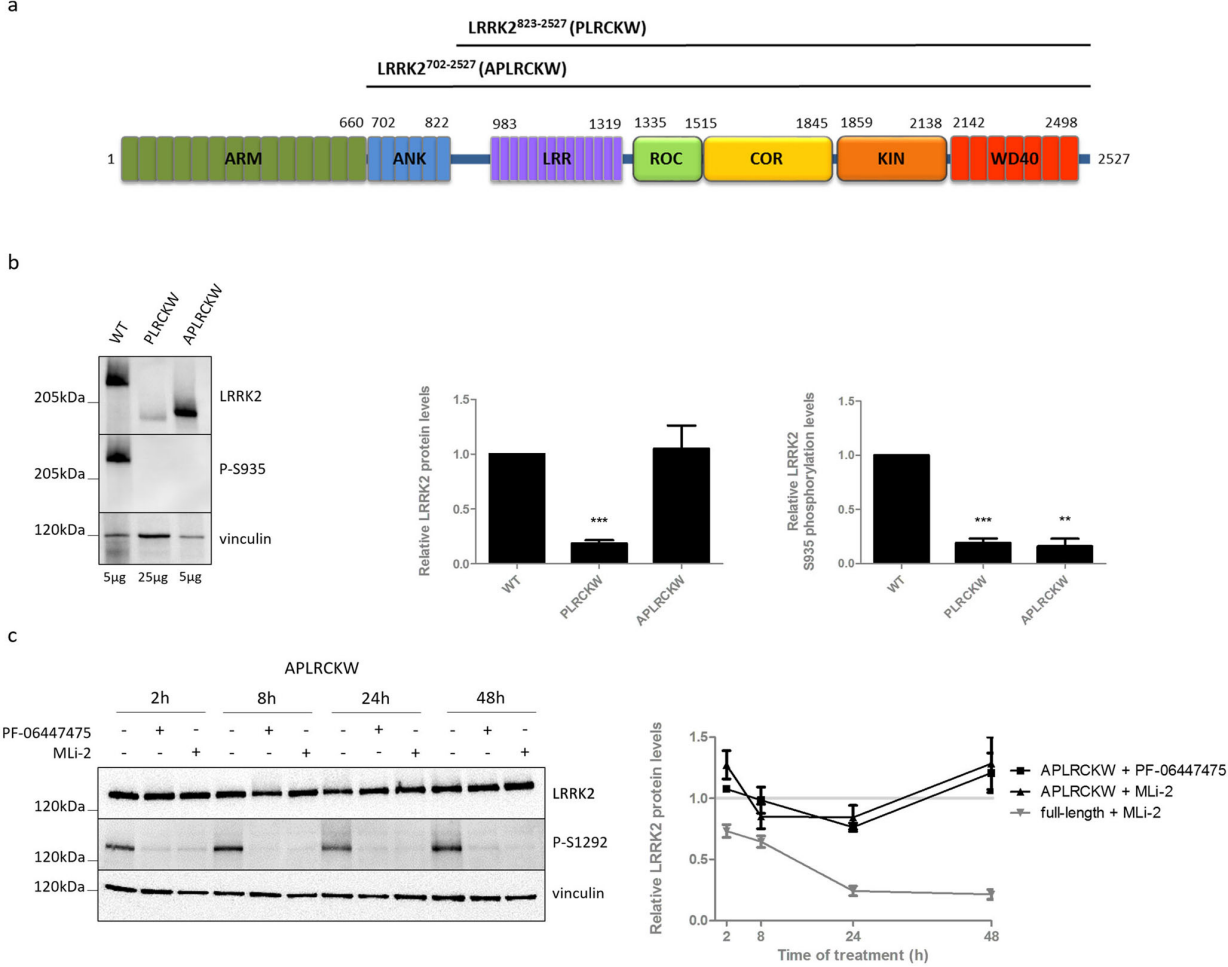
Protein levels of truncated forms of LRRK2 are not reduced upon LRRK2 kinase inhibitor treatment. We generated SH-SY5Y cells overexpressing 3Flag-truncated forms of LRRK2 (**a**): LRRK2^823-2527^ or PLRCKW, LRRK2^702-2527^, or APLRCKW. Cell lines were either not treated (**b**) or treated for different periods of time with PF-06447475 (150 nM), MLi-2 (10 nM), or DMSO as control (**c**). Cell lysates were analyzed with immunoblotting using FlagM2 antibody for LRRK2 detection, anti-LRRK2 P-S935, anti-LRRK2 P-S1292, or anti-vinculin for equal loading. Representative blots are shown. Graphs show the quantification of blots representing the ratio of total LRRK2 over housekeeping protein signal or the ratio of phosphorylation at S935 over total LRRK2 signal. Error bars indicate S.E.M. with *N* ≥ 3. Statistical significance was tested using a 2-way ANOVA test with Bonferroni post-tests or column statistics with Bonferroni correction. Triple asterisks indicate *p* < 0.001, double asterisks indicate *p* < 0.01

We have previously reported that, in contrast to full-length LRRK2, a ~ 170 kDa truncated form of LRRK2 in mouse kidney is dephosphorylated at S935, but not destabilized upon LRRK2 kinase inhibition [29]. Based on previous reports [30, 48] and the use of antibodies raised against different regions of LRRK2 (Fig. S1), we propose that this truncated form of LRRK2 lacks a part of the N-terminus. Therefore, we hypothesized that the N-terminal part of LRRK2 is involved in the regulation of LRRK2 protein degradation during kinase inhibition. We generated SH-SY5Y cells with stable overexpression of two truncated forms of LRRK2. The first one lacks the armadillo and ankyrin domain, but still contains the linker sequence N-terminal of the LRR that is highly phosphorylated in the full-length protein (LRRK2^823-2527^ or PLRCKW, predicated molecular weight of 193.8 kDa) (Fig. 2a). To investigate the importance of the ankyrin domain, we included a truncated LRRK2 variant only missing the armadillo domain (LRRK2^702-2527^ or APLRCKW, with predicted molecular weight of 206.9 kDa) (Fig. 2a). Remarkably, we were not able to detect basal phosphorylation at S935 in these truncated forms (Fig. 2b), suggesting that the N-terminal part of LRRK2 might be crucially involved in the regulation of LRRK2 phosphorylation at S935. Interestingly, treatment up to 48 h with PF-06447475 or MLi-2 did not induce a decrease in LRRK2 protein levels for both N-terminal truncated forms (Fig. 2c, Fig. S2b), while S1292 dephosphorylation confirmed inhibition of LRRK2 kinase activity (Fig. 2c). As an additional control for inhibitor activity, inhibition of LRRK2 kinase activity was assessed by S935 and S1292 dephosphorylation in parallel in a cell line expressing full-length LRRK2 WT (Fig. S2a).

### Several Pathogenic LRRK2 Mutants Do Not Destabilize upon LRRK2 Kinase Inhibition

**Fig. 3:**
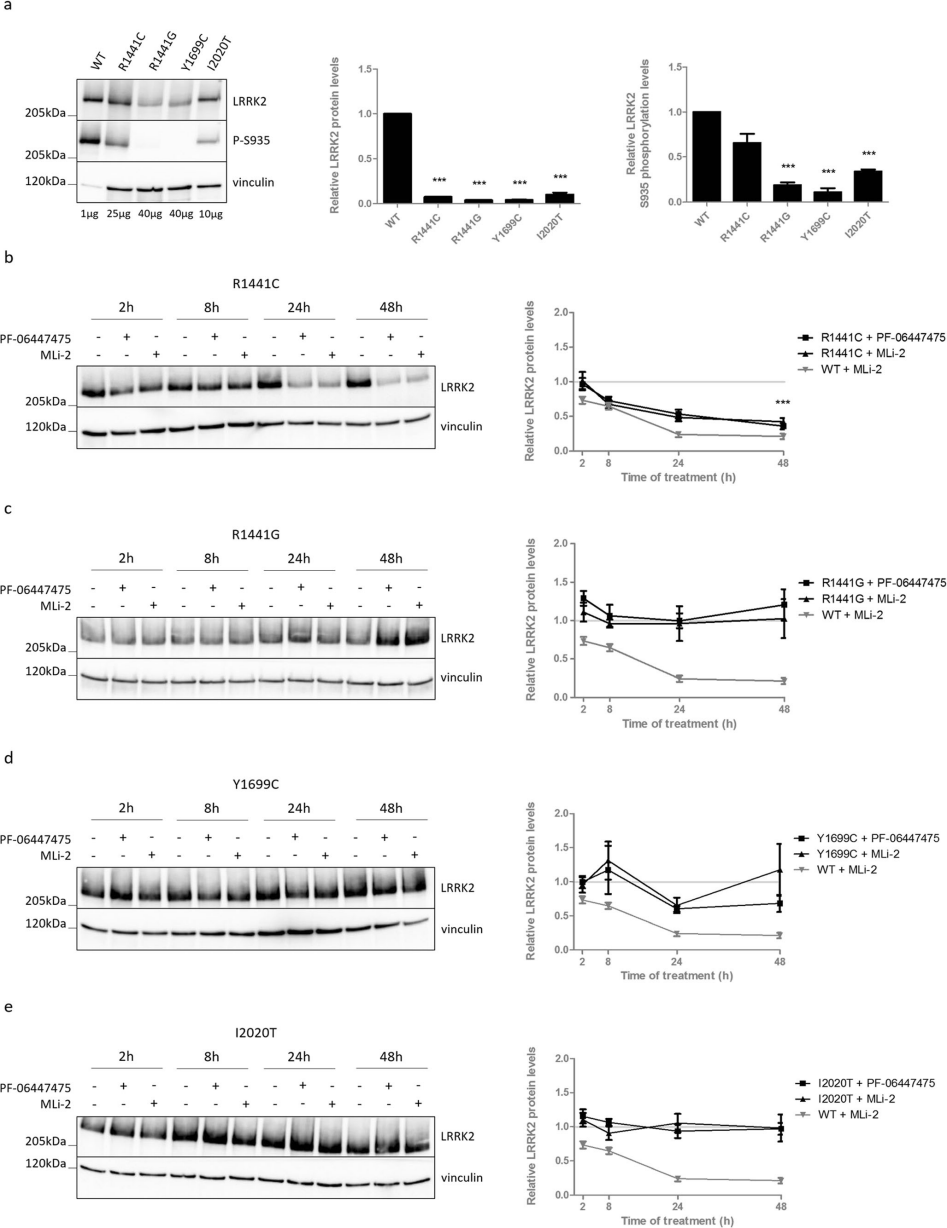
LRRK2 kinase inhibition does not induce destabilization in dephosphorylated pathogenic mutants. SH-SY5Y cells overexpressing LRRK2 R1441C, R1441G, Y1699C, or I2020T, were not treated (**a**) or treated for different periods of time with PF-06447475 (150 nM), MLi-2 (10 nM), or DMSO as control (**b–e**). Cell lysates were analyzed with immunoblotting using FlagM2 antibody for LRRK2 detection, anti-LRRK2 P-S935, or anti-vinculin. Representative blots are shown. Graphs show the quantification of blots representing the ratio of total LRRK2 over housekeeping protein signal or the ratio of phosphorylation at S935 over total LRRK2 signal. Error bars indicate S.E.M. with *N* ≥ 3. Statistical significance was tested using a 2-way ANOVA test with Bonferroni post-tests or column statistics with Bonferroni correction. Triple asterisks indicate *p* < 0.001

Several pathogenic LRRK2 mutations display strongly reduced expression levels and reduced basal phosphorylation at S935 (and other serines between the ankyrin and LRR domain) [49] (Fig. 3a). Therefore, we wondered whether these LRRK2 variants are still sensitive to LRRK2 kinase inhibitor-induced destabilization. Treatment of SH-SY5Y cells with stable overexpression of LRRK2 R1441G, Y1699C, or I2020T with two LRRK2 kinase inhibitors revealed that no LRRK2 protein degradation is induced in those variants with strongly reduced basal S935 phosphorylation levels (Fig. 3a, c–e). In contrast, LRRK2 R1441C, which only displays modest, non-significant, S935 dephosphorylation compared to LRRK2 WT under basal conditions (Fig. 3a), still destabilizes upon kinase inhibitor treatment (Fig. 3b). Again, WT LRRK2 was included in each of the experiments to confirm inhibition of LRRK2 kinase activity, as we could only confirm robust dephosphorylation at S1292 for LRRK2 R1441C and Y1699C (Fig. S3).

### Inhibition of Casein Kinase 1 Induces LRRK2 Destabilization

**Fig. 4:**
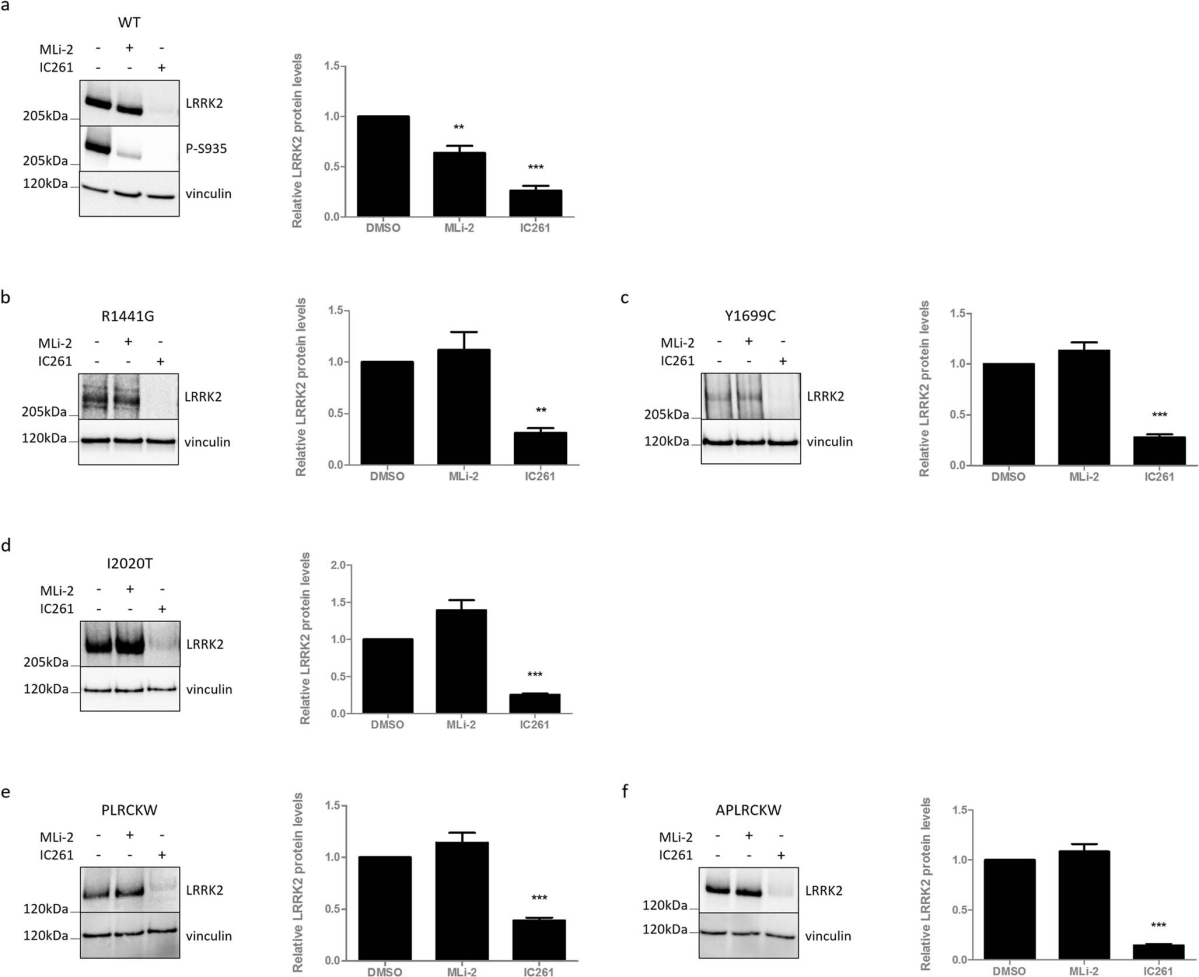
LRRK2 variants that do not destabilize upon LRRK2 kinase inhibition, do destabilize upon CK1 inhibition. SH-SY5Y cells overexpressing LRRK2 (**a**) WT, pathogenic: (**b**) R1441G, (**c**) Y1699C or (**d**) I2020T, truncated: (**e**) PLRCKW or (**f**) APLRCKW were treated between 6 and 8 h with MLi-2 (10 nM), IC261 (300 μM), or DMSO as control. Cell lysates were analyzed with immunoblotting using the FlagM2 antibody for LRRK2 detection, anti-LRRK2 P-S935, or anti-vinculin for equal loading. Representative blots are shown. Graphs show the quantification of blots representing the ratio of total LRRK2 over housekeeping proteins signal. Error bars indicate S.E.M. with *N* ≥ 3. Statistical significance was tested using column statistics with Bonferroni correction. Triple asterisks indicate *p* < 0.001, double asterisks indicate *p* < 0.01

The results with the clinical LRRK2 mutants suggest that heterologous phosphorylation levels rather than kinase activity of LRRK2 determine the inhibitor-induced destabilization effect. Chia et al. (2014) identified casein kinase 1α (CK1α) as the kinase responsible for phosphorylation of S935 [44]. Therefore, we decided to inhibit the upstream LRRK2 kinase CK1α in cells expressing LRRK2 WT and to examine the effect on LRRK2 protein stability (Fig. 4a). Six hours of treatment with the CK1 inhibitor IC261 induced LRRK2 S935 dephosphorylation, as described previously [44]. This was accompanied by a strong reduction in total LRRK2 protein levels, which was even more pronounced than after MLi-2 treatment (Fig. 4a). This might indicate that the LRRK2 phosphorylation levels and not the kinase activity per se are important for the destabilization effect. Next, we examined the effect of CK1 inhibition on LRRK2 variants that are not destabilized after LRRK2 kinase inhibition. Interestingly, protein levels of pathogenic mutants R1441G (Fig. 4b), Y1699C (Fig. 4c), and I2020T (Fig. 4d), as well as the two truncated LRRK2 variants (lacking the armadillo domain or/and the ankyrin domain) (Fig. 4e, f), were significantly decreased after treatment with CK1 inhibitor, but not with LRRK2 kinase inhibitor.

### Known Heterologous Phosphorylation Sites Do Not Determine LRRK2 Destabilization

**Fig. 5:**
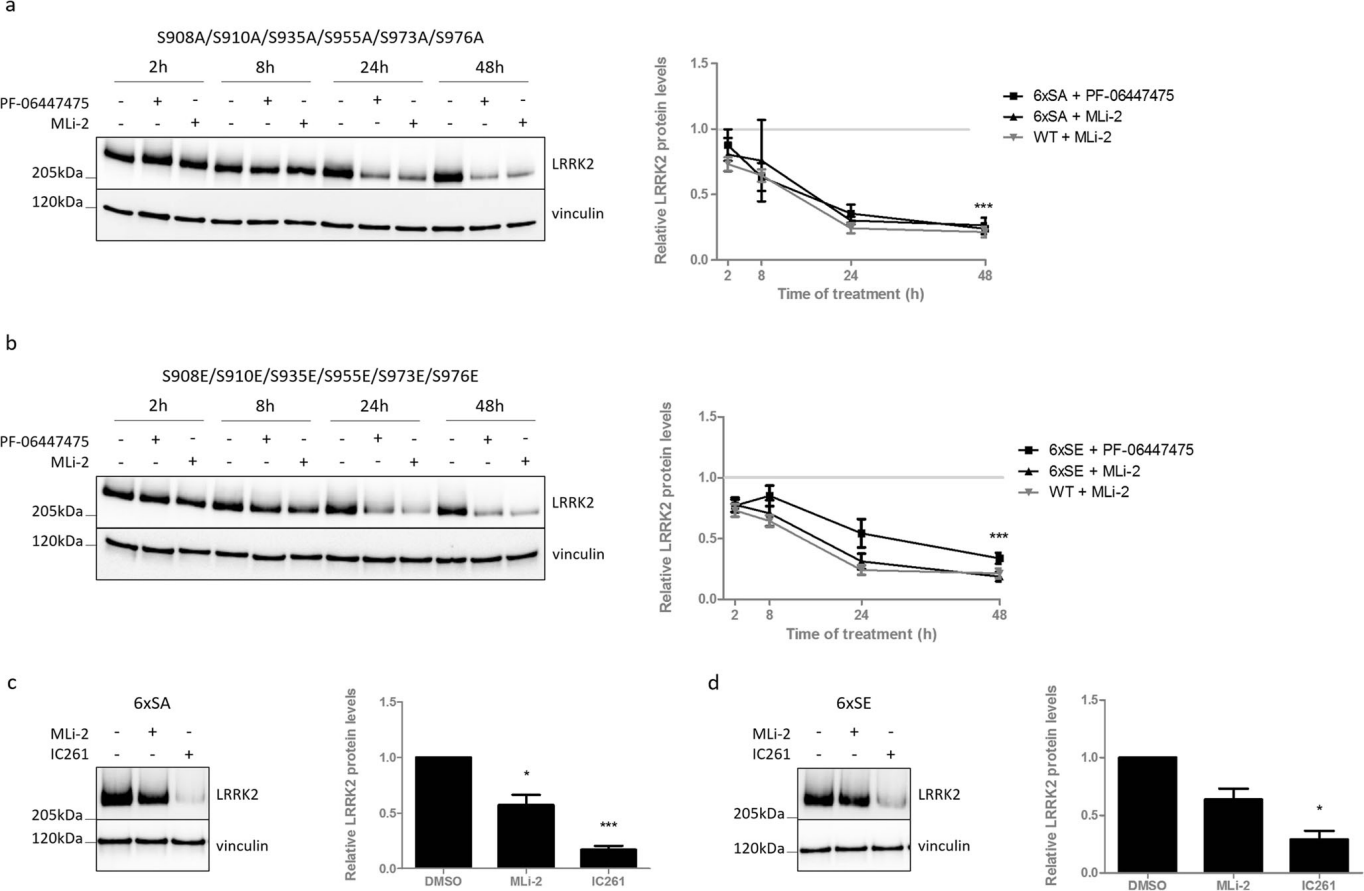
Dephosphorylation at residues S908, S910, S935, S955, S973, or S976 is not crucial for LRRK2 kinase inhibitor-induced destabilization. SH-SY5Y cells overexpressing LRRK2 with (**a**, **c**) 6xSA (S908A/S910A/S935A/S955A/S973A/S976A) or (**b**, **d**) 6xSE (S908E/S910E/S935E/S955E/S973E/S976E) were treated (**a**, **b**) for different periods of time with PF-06447475 (150 nM), MLi-2 (10 nM), or DMSO as control or (**c**, **d**) between 6 and 8 h with MLi-2 (10 nM), IC261 (300 μM), or DMSO as control. Cell lysates were analyzed with immunoblotting using FlagM2 antibody for LRRK2 detection or anti-vinculin for equal loading. Representative blots are shown. Graphs show the quantification of blots representing the ratio of total LRRK2 over housekeeping protein signal. Error bars indicate S.E.M. with *N* ≥ 3. Statistical significance was tested using a 2-way ANOVA test with Bonferroni post-tests or column statistics with Bonferroni correction. Triple asterisks indicate *p* < 0.001, asterisk indicates *p* < 0.05

The results obtained so far might indicate that the basal phosphorylation at S935 is crucial for the inhibitor-induced LRRK2 destabilization, as was suggested previously [28]. However, we previously reported that a truncated form of LRRK2 in mouse kidney could be dephosphorylated at S935, but not destabilized [29]. In addition, substitution to an alanine at S935 or S910 could not prevent LRRK2 from degradation during kinase inhibition [29], indicating that S935 dephosphorylation is not sufficient for inhibitor-induced LRRK2 destabilization. In fact, S935 is part of a cluster of heterologous phosphorylation sites N-terminal of the LRR domain, and CK1 has been reported to phosphorylate not only S935, but also the S910, S955, and S973 sites, in addition to two extra residues, S908 and S976. Therefore, we hypothesized that dephosphorylation of one of the other serines might be involved in the destabilization effects induced by pharmacological LRRK2 kinase inhibition. To test this, we generated SH-SY5Y cells stably overexpressing a mutant form of LRRK2, in which the six serines are mutated to an alanine (phosphodeficient) or glutamic acid (phosphomimetic). Treatment of these cell lines with PF-06447475 or MLi-2 revealed that the 6× phosphodeficient and phosphomimetic mutant LRRK2 still destabilized upon LRRK2 kinase inhibition to the same extent as WT LRRK2 (Fig. 5a, b). Remarkably, the phosphodeficient LRRK2 variant displays increased autophosphorylation levels at S1292 after LRRK2 kinase inhibition, while effects on autophosphorylation levels after LRRK2 kinase inhibition of the phosphomimetic LRRK2 variant are elusive (Fig. S4). Also, in this set of experiments, cell lines overexpressing WT LRRK2 were treated in parallel and confirmed inhibitor-induced dephosphorylation at S1292 and S935, as expected.

Treatment of these mutants with CK1 inhibitor resulted in a stronger reduction in total LRRK2 protein levels compared to LRRK2 kinase inhibition, indicating that dephosphorylation at the six phosphorylation sites is not essential for inhibitor-induced degradation of LRRK2 (Fig. 5c, d).

### CK1 Inhibition Induces LRRK2 Protein Destabilization in the Lung

Next, we aimed to investigate the effect of CK1 inhibition in a more physiological model. Remarkably, LRRK2 kinase inhibitor or CK1 inhibitor treatment of primary cortical neurons did not induce significant changes in LRRK2 protein levels (Fig. S5). It should be noted that IC261 treatment induced cellular toxicity upon 8 h of treatment, which might explain the lack of effect on LRRK2. Next, we examined the effect of CK1 inhibition in vivo*.* Since little information is available on brain permeability and differences in potency and isoform-specificity have been ascribed to different CK1 inhibitors [50], we compared two CK1 inhibitors, IC261 and PF-670462. Since PF-670462 induced the strongest LRRK2 protein destabilization and this compound has been reported to have a greater potency to inhibit CK1 compared to IC261 [50], PF-670462 was selected for further in vivo experiments.

**Fig. 6:**
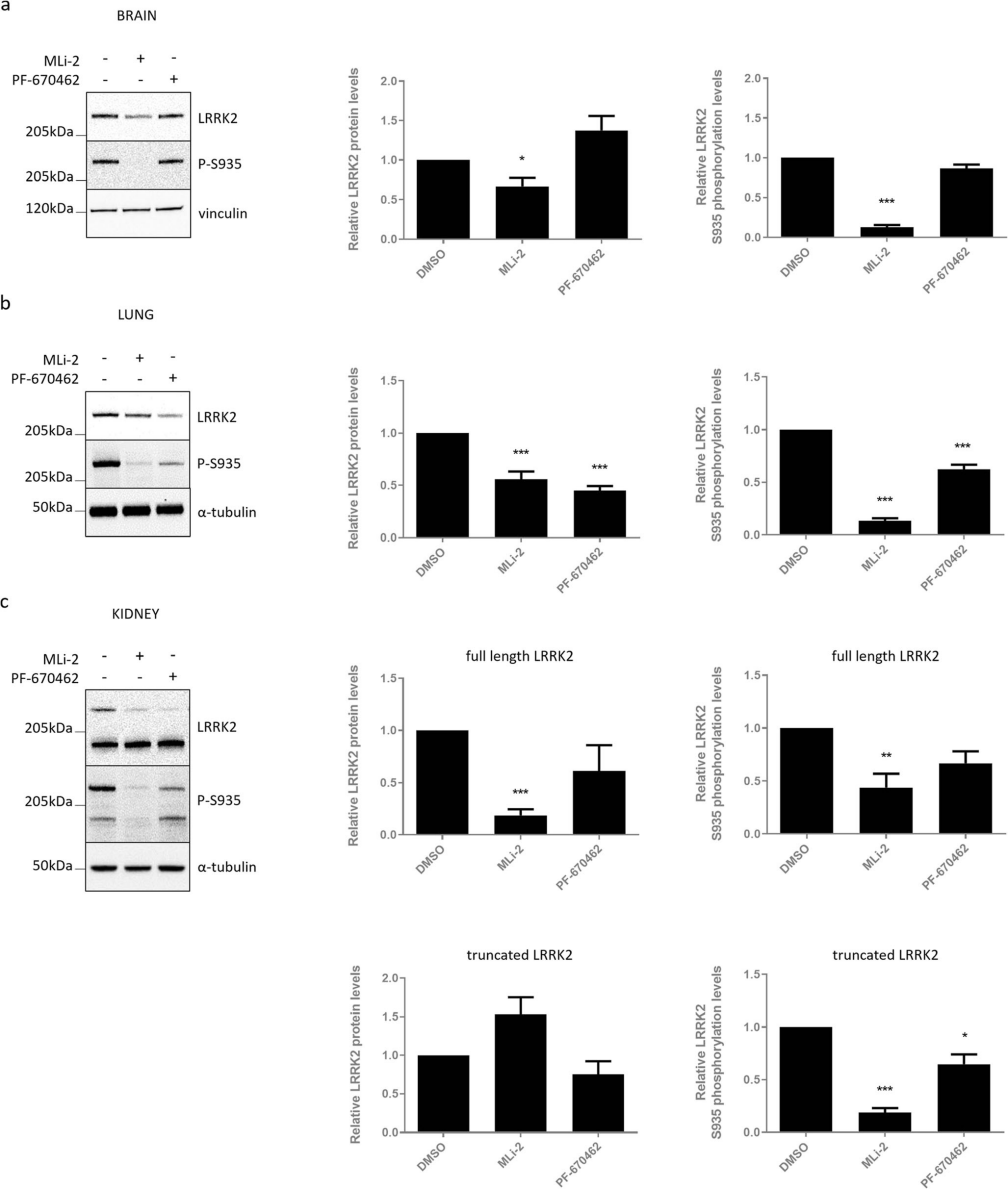
CK1 inhibition-induced destabilization of LRRK2 protein also occurs in vivo. C57Bl/6J mice were injected four times intraperitoneally with the LRRK2 kinase inhibitor MLi-2 (10 mg/kg), the CK1 inhibitor PF-670462 (50 mg/kg), or DMSO as a negative control over a period of 30 h. Brain (**a**), lung (**b**), and kidney (**c**) extracts were taken 2 h after the last injection and analyzed with immunoblotting using MJFF-2 anti-LRRK2 antibody, anti-LRRK2 P-S935, or anti-α-tubulin or anti-vinculin for equal loading. Representative blots are shown. Graphs show the quantification of blots representing the ratio of total LRRK2 signal over housekeeping protein signal or the ratio of phosphorylation at S935 over total LRRK2 signal. Error bars indicate S.E.M. with *N* ≥ 3. Statistical significance was tested using column statistics with Bonferroni correction. Triple asterisks indicate *p* < 0.001, double asterisks indicate *p* < 0.01, asterisk indicates *p* < 0.05

We treated wild-type mice with the LRRK2 kinase inhibitor MLi-2 (10 mg/kg), CK1 inhibitor PF-670462 (50 mg/kg), or with DMSO, and analyzed brain, lung, and kidney tissue. As shown previously [29], LRRK2 kinase inhibition induced a significant decrease in LRRK2 phosphorylation at S935 and total full-length LRRK2 protein levels in the brain, lung, and kidney (Fig. 6a–c). In contrast, protein stability of the truncated LRRK2 variant in the kidney was not affected, despite a significant dephosphorylation at S935 (Fig. 6c). CK1 inhibition induced LRRK2 S935 dephosphorylation in lung and kidney; however, no S935 dephosphorylation could be observed in brain extracts. In line with the cellular experiments, CK1 inhibition induced a significant reduction in total LRRK2 protein levels in the lung (Fig. 6b).

## Discussion

How LRRK2 proteostasis is regulated in basal conditions and upon pharmacological kinase inhibition is not completely understood. Since clinical applications will most likely require long-term administration and thus a chronic suppression of LRRK2 protein levels, we have investigated the effects of sustained LRRK2 kinase inhibition in the present study. We found that LRRK2 S935 dephosphorylation and total protein destabilization are maintained during chronic LRRK2 kinase inhibition, but are reversible when the inhibitor is withdrawn. This is in line with the finding that the lung phenotype, observed in non-human primates treated with different LRRK2 kinase inhibitors, is normalized upon cessation of compound administration [51]. Therefore, clinical studies with long-term dosing, ideally in a Parkinson’s disease context, will have to reveal to what extent the observed phenotypes are a real safety issue.

Here, we confirm our previously reported finding that a physiologically occurring truncated LRRK2 form in mouse kidney, which still contains the S935, the LRR, COR, and kinase domain (Fig. S1 and [29, 30]), does not destabilize upon LRRK2 kinase inhibition in contrast to full-length LRRK2 (Fig. 6 and [29]). Using truncated forms of LRRK2, we here show that the presence of the armadillo domain, or N-terminus of LRRK2, is crucial for inhibitor-induced LRRK2 protein degradation. As these LRRK2 variants are artificial mutants, we cannot rule out potential effects on protein folding and hence induction of secondary effects. For example, the epitope for the P-S935 antibody might be inaccessible in these truncated variants, explaining the lack of phosphorylated S935 detection. Still, our findings are in line with the observations on the truncated form in the kidney. A ~ 170 kDa truncated form of LRRK2 was also observed in neutrophils and could be destabilized by 30 min of MLi-2 treatment, which was not the case for its full-length counterpart [48]. These apparently contradicting results might be related to cell type-specific effects [29], different sequences of this truncation, or an alternative mechanism, as high doses of another specific inhibitor, PF-06447475, could not induce a similar effect in neutrophils.

Dephosphorylation of S935 is induced by every LRRK2 kinase inhibitor reported to date and was previously suggested to be sufficient for LRRK2 degradation [28]. However, our findings point to a much more complex mechanism as the truncated form of LRRK2 in mouse kidney can still be dephosphorylated at S935, but is not destabilized upon LRRK2 kinase inhibition [29]. By extension, using a phosphodeficient and phosphomimetic mutant, we show that none of the six well-characterized heterologous phosphorylation sites (i.e., S908, S910, S935, S955, S973, and S976) are crucially involved in inhibitor-induced LRRK2 protein degradation. To our knowledge, these functional LRRK2 mutants have not been reported before. The intriguing observation that the phosphodeficient LRRK2 variant displays increased autophosphorylation at S1292 after LRRK2 kinase inhibition might point to an interesting regulatory mechanism and will need additional investigation. Still, we should take into account that the introduction of artificial phosphorylation site mutations might affect the conformation, resulting in secondary molecular changes.

In contrast to LRRK2 G2019S, which is destabilized by kinase inhibition to the same extent as LRRK2 WT [29], pathogenic mutations R1441G, Y1699C, and I2020T are not degraded when inhibited. This is in line with the reported lack of increased ubiquitination upon LRRK2 kinase inhibition for LRRK2 mutants R1441G, Y1699C, and I2020T [28]. Of particular interest is the finding that the R1441C variant behaves similarly to LRRK2 WT in terms of inhibitor-induced destabilization. Remarkably, LRRK2 R1441C only displays a modest reduction in basal phosphorylation compared to LRRK2 WT, in contrast to the pathogenic variants that are not destabilized upon inhibition and show strongly reduced heterologous phosphorylation [49]. Also, truncated LRRK2 (i.e., both the artificial constructs used here and the physiologically occurring LRRK2 truncation in the kidney) does not destabilize and displays strongly reduced heterologous phosphorylation (at least at S935) [29]. These data suggest that pharmacological LRRK2 kinase inhibition is not sufficient for LRRK2 protein destabilization and point to dephosphorylation of LRRK2 as a potential mechanism underlying inhibitor-induced destabilization, albeit not at S908, S910, S935, S955, S973, or S976. Although future studies should confirm these findings on endogenous (knock-in) LRRK2, the observation that several pathogenic mutants are not degraded after LRRK2 kinase inhibition is intriguing and should be taken into consideration when stratifying clinical studies. Indeed, potential protective effects of kinase inhibitors might be mutation-dependent and not only rely on the decrease in kinase activity, but also on the degree of protein degradation, as shown in cell lines [31].

To further investigate the hypothesis that phosphorylation levels rather than kinase activity of LRRK2 determines the inhibitor-induced destabilization effect, we assessed the effects of inhibition of CK1, which was reported as an upstream LRRK2 kinase [44]. Interestingly, pharmacological CK1 inhibition led to a strong and fast reduction in LRRK2 protein levels. Therefore, we here identify CK1 as a new regulator of LRRK2 homeostasis in cell culture and in vivo. As CK1 inhibition can induce protein destabilization in LRRK2 variants that are insensitive to LRRK2 kinase inhibitor-induced destabilization, we reasoned that CK1 might act downstream of LRRK2 kinase inhibition in the pathway that ultimately leads to LRRK2 degradation. Somewhat surprisingly, using a sixfold phosphorylation mutant of LRRK2, none of the reported LRRK2 target sites of CK1α appeared to be crucially involved in LRRK2 kinase inhibitor-induced destabilization. This corresponds to our observation that protein levels of these phosphorylation deficient/mimetic variants are still reduced upon CK1 inhibition. Since the CK1 inhibitors used here are not specific for the CK1α isoform, which was proposed as a LRRK2 interactor [44], we cannot rule out that other CK1 isoforms are involved in the regulation of LRRK2 protein stability in different tissues. Indeed, the CK1δ and ε isoform preference of CK1 inhibitor PF-670462 might explain the lack of LRRK2 S935 dephosphorylation in brain tissue, although low brain permeability of the compound cannot be excluded either.

In the kidney, we observed a trend towards decreased LRRK2 protein levels for full-length LRRK2 and truncated LRRK2, although S935 dephosphorylation of full-length LRRK2 did not reach significance (Fig. 6c). In contrast, we have observed strong destabilization of artificially truncated LRRK2 variants after CK1 inhibition in cell culture. An important consideration is the similarity between our artificial truncated variant and the one found in the kidney as this might help to explain our observations.

Although more research is required to exclude indirect or kinase-independent effects of CK1, we postulate that inhibition of CK1 induces LRRK2 dephosphorylation at yet unidentified sites, which ultimately induces proteasomal degradation. The hypothesis of dephosphorylation-induced destabilization of LRRK2 is in line with the findings of Zhao et al. that inhibition of 14-3-3 binding by difopein treatment reduces LRRK2 protein levels, taking into account that 14-3-3 proteins interact with LRRK2 in a phosphorylation-dependent manner. In addition, they showed that inhibition of protein phosphatase 1 and 2A prevents increased ubiquitination induced by pharmacological kinase inhibition [28]. Given the evidence that phosphorylation of LRRK2 is involved in inhibitor-induced LRRK2 protein destabilization, a potential role for other upstream LRRK2 phosphorylation regulators such as PKA, PAK6, and IKKα/β (summarized in [52], reviewed in [53]) should be considered in future research in addition to CK1.

Taken together, we hypothesize that pharmacological LRRK2 kinase inhibition induces N-terminal changes that lead to reduced CK1 interaction, resulting in reduced heterologous phosphorylation and ultimately LRRK2 protein degradation. This is in line with the reported close interactions between the N-terminal part of LRRK2 (mainly the ankyrin domain) and its kinase domain [54], as well as with the reduced LRRK2-CK1 interaction after LRRK2 kinase inhibition [44]. To date, it is not clear which phosphorylation sites are involved in this process, although they probably interact with 14-3-3 proteins, and are located outside the N-terminal part of LRRK2, given that also truncated LRRK2 variants destabilized upon CK1 inhibition.

Finally, the clinical LRRK2 mutants R1441G, Y1699C, and I2020T as well as the artificial N-terminal LRRK2 truncations display reduced expression levels in all stable cell lines generated (Figs. 2b and 3a), which might explain why phosphorylation levels at S1292 were under the detection limit for some of these LRRK2 variants. The reduced expression levels might be explained by the reported reduction in half-life of pathogenic LRRK2 mutants compared to LRRK2 WT [45, 55, 56]. As these LRRK2 variants also display reduced phosphorylation levels at S935, a similar pathway as hypothesized above may underlie the reduced protein stability in basal conditions. This would involve decreased interaction of pathogenic mutants or N-terminal truncations with CK1, leading to basally dephosphorylated residues, thereby marking LRRK2 for protein degradation.

In conclusion, we show that chronic treatment with LRRK2 kinase inhibitors induces sustained LRRK2 S935 dephosphorylation and LRRK2 degradation, which can be reversed after inhibitor withdrawal. We identify the N-terminus as a crucial mediator of inhibitor-induced protein degradation. We show that S935 dephosphorylated pathogenic mutations R1441G, Y1699C, and I2020T do not destabilize upon LRRK2 kinase inhibition, in contrast to LRRK2 WT or R1441C. Lastly, inhibition of CK1 induces fast and strong destabilization of LRRK2, also in conditions where LRRK2 kinase inhibitor cannot. The involvement of CK1 in the regulation of endogenous LRRK2 protein levels was further confirmed in vivo. Altogether, we postulate that LRRK2 kinase inhibition induces N-terminal changes that disrupt the LRRK2-CK1 interaction, leading to reduced heterologous phosphorylation and LRRK2 protein degradation. Although several questions remain, our study provides important new insights in LRRK2 protein homeostasis.

## Electronic supplementary material

Isolation and culture of primary neurons. All animal experiments were performed in accordance with the European Communities Council Directive of November 24, 1986 (86/609/EEC) and approved by the Bioethical Committee of the KU Leuven (Belgium). Primary cortical neurons were prepared from newborn (P0) FVB pups. Cells were dissociated by 15 min incubation at 37 °C using trypsin (Gibco), followed by physical dissociation bypipetting. Cells were plated in a 6-well plate precoated with poly-D-lysine (Sigma) in Neurobasal medium (Gibco) supplemented with B27 and 2 mM L-glutamine. Seven days later, cells were treated for 48 h with 10nMMLi-2 or DMSO or for 8 h with 300 μM IC261. Cells were lysed with 1% SDS supplemented with protease and phosphatase inhibitors (Roche) and subjected to immunoblot analysis.

ᅟ

Fig. S2 SH-SY5Y cells with overexpression of LRRK2 WT (**a**) or PLRCKW (**b**) were treated with LRRK2 kinase inhibitors PF-06447475 (150 nM), MLi-2 (10 nM) or DMSO as control for several periods of time. Cell lysates were analyzed with immunoblotting using FlagM2 antibody for LRRK2 detection, anti-LRRK2 P-S935, anti-LRRK2 P-S1292 or anti-vinculin for equal loading. Representative blots are shown. The graph shows the quantification of blots representing the ratio of total LRRK2 over housekeeping protein signal. Error bars indicate S.E.M. with *N* ≥ 3.Statistical significance was tested using a 2-way ANOVA test with a Bonferroni post-test. (PNG 208 kb)

High Resolution Image (PNG 62 kb) (TIF 13004 kb)

Fig. S3 SH-SY5Y cells with overexpression of LRRK2 R1441C (**a**), R1441G (**b**), Y1699C (**c**) or I2020T (**d**) were treated with LRRK2 kinase inhibitors PF-06447475 (150 nM), MLi-2 (10 nM) or DMSO as control for several periods of time. Cell lysates were analyzed with immunoblotting using FlagM2 antibody for LRRK2 detection, anti-LRRK2 P-S1292 or anti-vinculin. (PNG 235 kb)

High Resolution Image (TIF 11502 kb)

Fig. S4 SH-SY5Y cells with overexpression of phosphomutant LRRK2 S908A/S910A/S935A/S955A/S973A/S976A (**a**) or S908E/S910E/S935E/S955E/S973E/S976E (**b**) were treated with LRRK2 kinase inhibitors PF-06447475 (150 nM), MLi-2 (10 nM) or DMSO as control for several periods of time. Cell lysates were analyzed with immunoblotting using FlagM2 antibody for LRRK2 detection, anti-LRRK2 P-S1292 or anti-vinculin. (PNG 354 kb)

High Resolution Image (TIF 20735 kb)

Fig. S5 Primary cortical neurons were treated for 48 h with MLi-2 (10 nM) or 8 h with IC261 (300 μM). DMSO was used as negative control. Cell lysates were analyzed with immunoblotting using MJFF-2 antibody for LRRK2 detection, anti-LRRK2 P-S935 or anti-vinculin for equal loading. Graphs show the quantification of blots representing the ratio of total LRRK2 over housekeeping proteins or the ratio of phosphorylation at S935 over total LRRK2 signal. Error bars indicate S.E.M. with *N* ≥ 3. Statistical significance was tested using column statistics with Bonferroni correction. * *p* < 0.05. (PNG 62 kb)

High Resolution Image (TIF 4987 kb)

Supplemental Figure 1 The ~170kDa truncated LRRK2 variant in the kidney lacks an N-terminal part. Cell lysates from kidney tissue (C57Bl/6J WT or LRRK2-/- mice) were analyzed with immunoblotting using anti-LRRK2 MJFF-2 (ab133474), anti-LRRK2/Dardarin, N-terminus N138/6 (Neuromab 75-188), anti-LRRK2/Dardarin, Cterminus N241A/34 (Neuromab 75-253), anti-LRRK2 MC.028.83.76.242 (ab130277). The arrow indicates the presumed truncated LRRK2 variant (JPEG 257 kb)

## Abbreviations

APLRCKW: truncated form of LRRK2 which lacks the armadillo domain
BCA: bicinchoninic acid assay
CK: casein kinase
COR: C-terminal of ROC
DMEM: Dulbecco modified eagle medium
EDTA: ethylenediaminetetraacetic acid
LRRK2: leucine-rich repeat kinase 2
LV: lentiviral
PAK6: p21-activated kinase 6
PBS: phosphate-buffered saline
PBS-T: phosphate-buffered saline supplemented with 0.1% Triton X-100
PD: Parkinson’s disease
PLRCKW: truncated form of LRRK2 which lacks both the armadillo and the ankyrin domain
PP1: protein phosphatase 1
ROC: Ras of complex proteins
WT: wild type
