# Supplementary figures and images for: Inhibition of LRRK2 or Casein Kinase 1 Results in LRRK2 Protein Destabilization
Source: Mol Neurobiol. 2018 Dec 27;56(8):5273–86. doi: 10.1007/s12035-018-1449-2 (PMC6657425; doi:10.1007/s12035-018-1449-2)

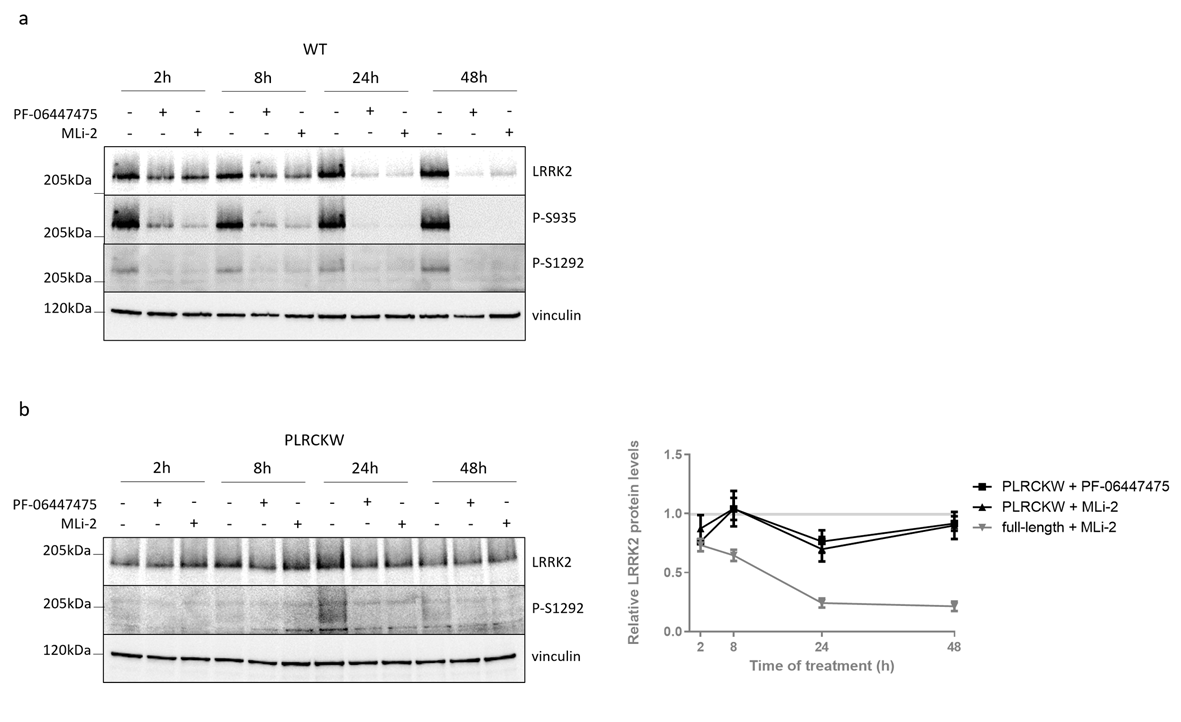

Supplement: Supplementary file 1 — SH-SY5Y cells with overexpression of LRRK2 WT (a) or PLRCKW (b) were treated with LRRK2 kinase inhibitors PF-06447475 (150 nM), MLi-2 (10 nM) or DMSO as control for several periods of time. Cell lysates were analyzed with immunoblotting using FlagM2 antibody for LRRK2 detection, anti-LRRK2 P-S935, anti-LRRK2 P-S1292 or anti-vinculin for equal loading. Representative blots are shown. The graph shows the quantification of blots representing the ratio of total LRRK2 over housekeeping protein signal. Error bars indicate S.E.M. with N ≥ 3.Statistical significance was tested using a 2-way ANOVA test with a Bonferroni post-test. (PNG 208 kb) [file 12035_2018_1449_Fig7_ESM.png]

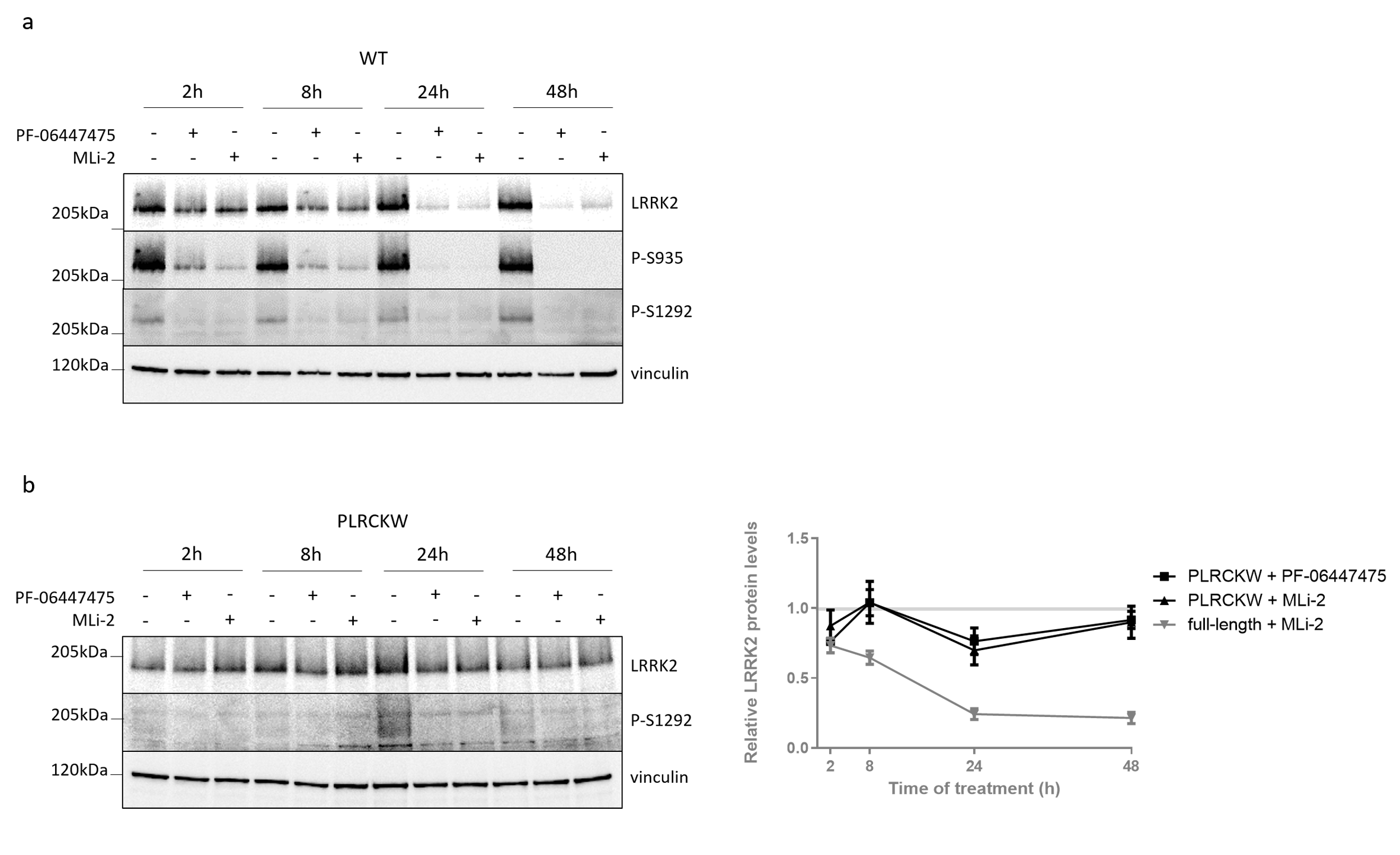

Supplement: Supplementary file 2 — High Resolution Image (PNG 62 kb) (TIF 13004 kb) [file 12035_2018_1449_MOESM1_ESM.tif]

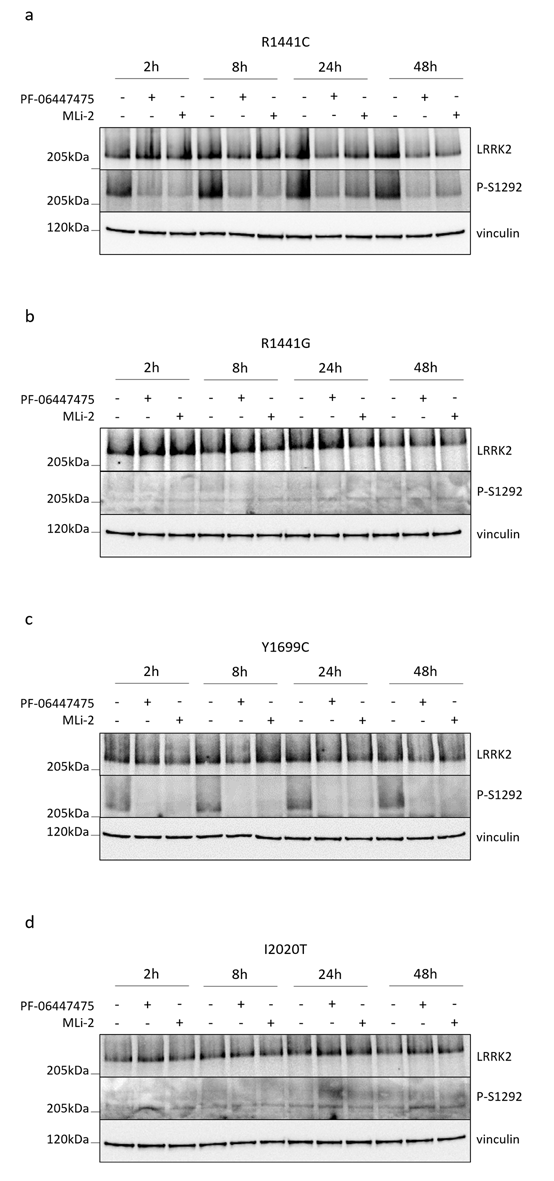

Supplement: Supplementary file 3 — SH-SY5Y cells with overexpression of LRRK2 R1441C (a), R1441G (b), Y1699C (c) or I2020T (d) were treated with LRRK2 kinase inhibitors PF-06447475 (150 nM), MLi-2 (10 nM) or DMSO as control for several periods of time. Cell lysates were analyzed with immunoblotting using FlagM2 antibody for LRRK2 detection, anti-LRRK2 P-S1292 or anti-vinculin. (PNG 235 kb) [file 12035_2018_1449_Fig8_ESM.png]

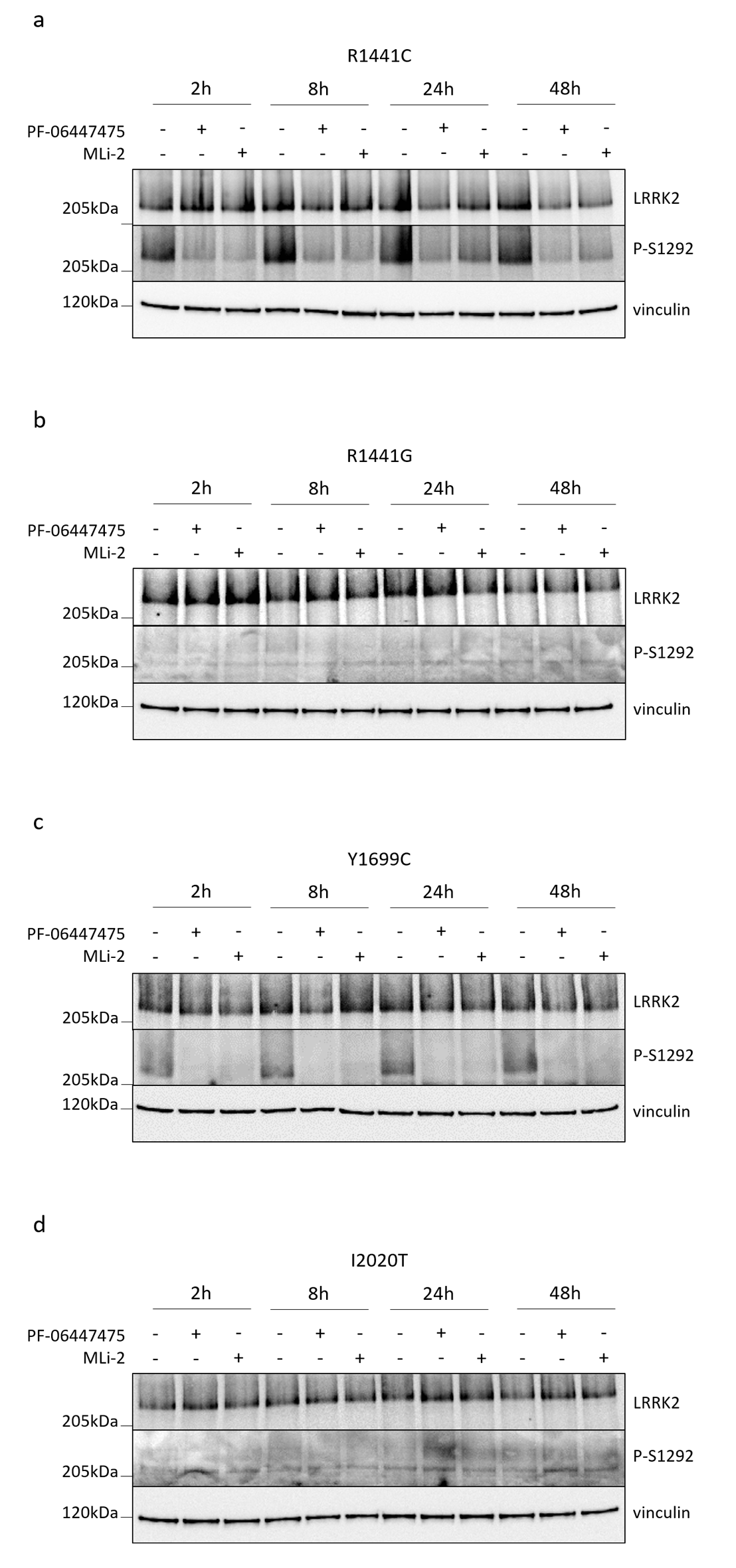

Supplement: Supplementary file 4 — High Resolution Image (TIF 11502 kb) [file 12035_2018_1449_MOESM2_ESM.tif]

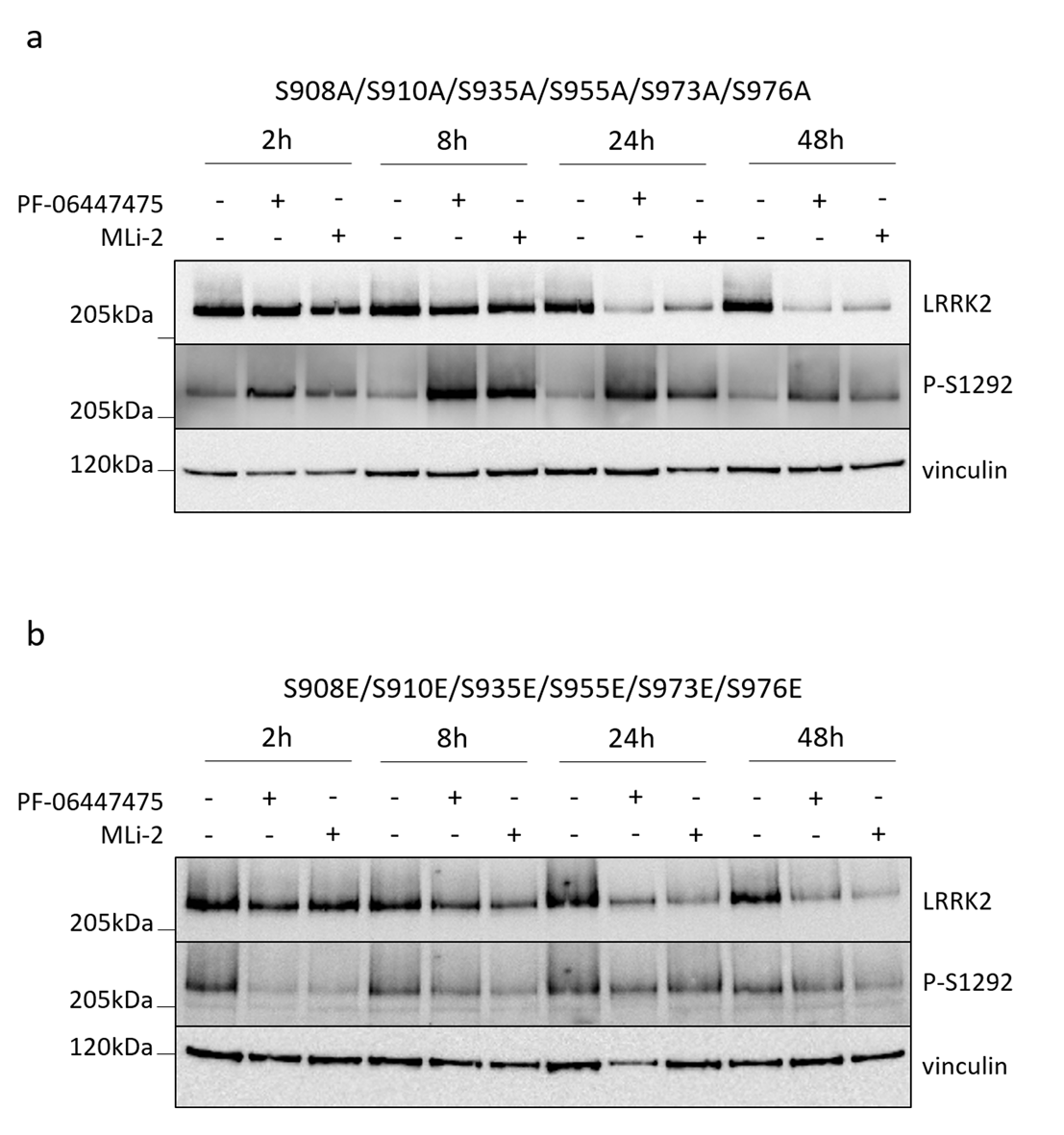

Supplement: Supplementary file 5 — SH-SY5Y cells with overexpression of phosphomutant LRRK2 S908A/S910A/S935A/S955A/S973A/S976A (a) or S908E/S910E/S935E/S955E/S973E/S976E (b) were treated with LRRK2 kinase inhibitors PF-06447475 (150 nM), MLi-2 (10 nM) or DMSO as control for several periods of time. Cell lysates were analyzed with immunoblotting using FlagM2 antibody for LRRK2 detection, anti-LRRK2 P-S1292 or anti-vinculin. (PNG 354 kb) [file 12035_2018_1449_Fig9_ESM.png]

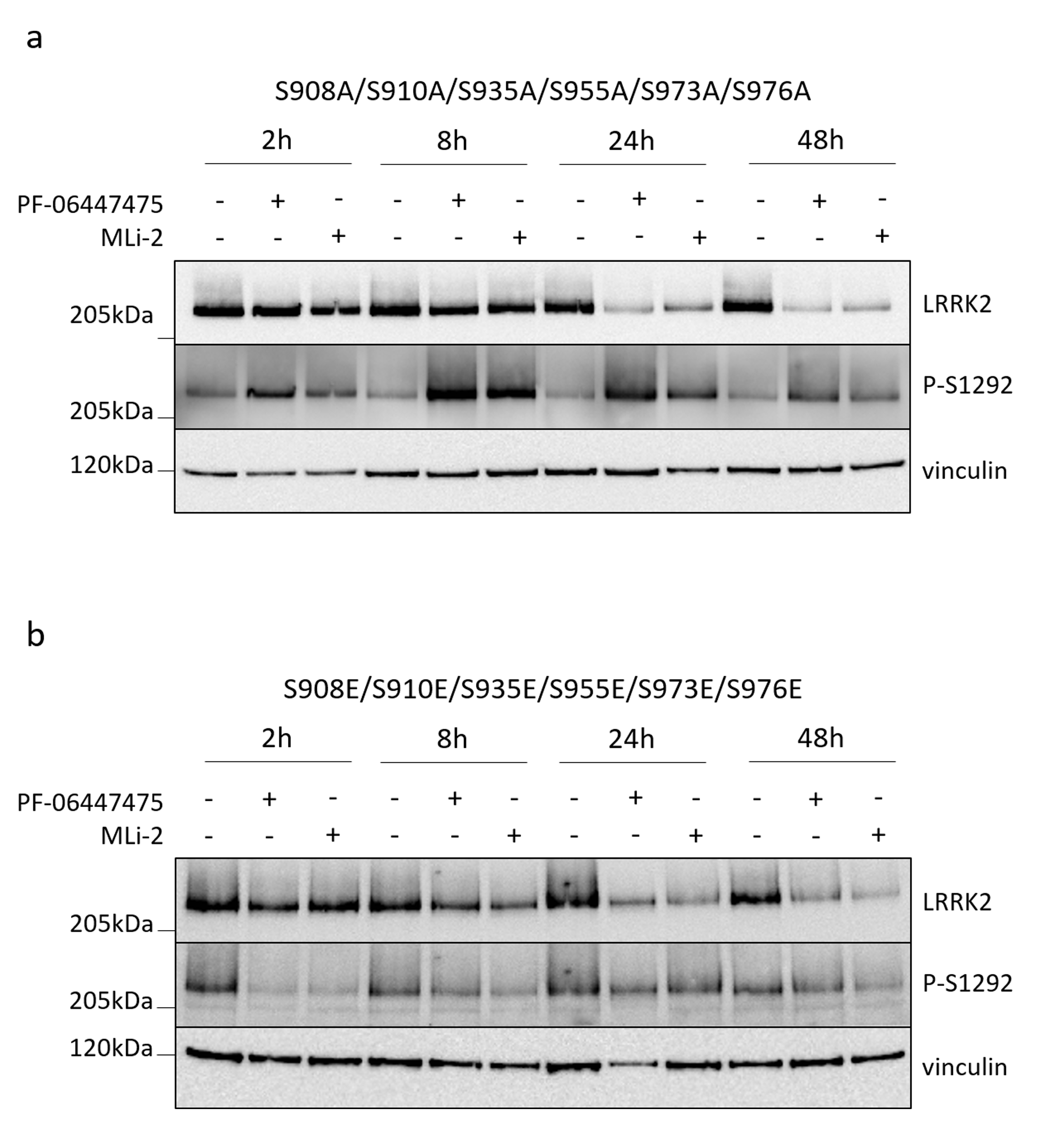

Supplement: Supplementary file 6 — High Resolution Image (TIF 20735 kb) [file 12035_2018_1449_MOESM3_ESM.tif]

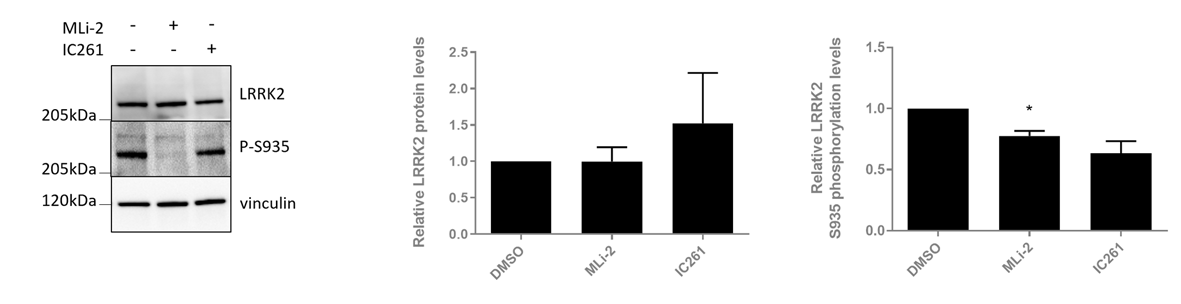

Supplement: Supplementary file 7 — Primary cortical neurons were treated for 48 h with MLi-2 (10 nM) or 8 h with IC261 (300 μM). DMSO was used as negative control. Cell lysates were analyzed with immunoblotting using MJFF-2 antibody for LRRK2 detection, anti-LRRK2 P-S935 or anti-vinculin for equal loading. Graphs show the quantification of blots representing the ratio of total LRRK2 over housekeeping proteins or the ratio of phosphorylation at S935 over total LRRK2 signal. Error bars indicate S.E.M. with N ≥ 3. Statistical significance was tested using column statistics with Bonferroni correction. * p < 0.05. (PNG 62 kb) [file 12035_2018_1449_Fig10_ESM.png]

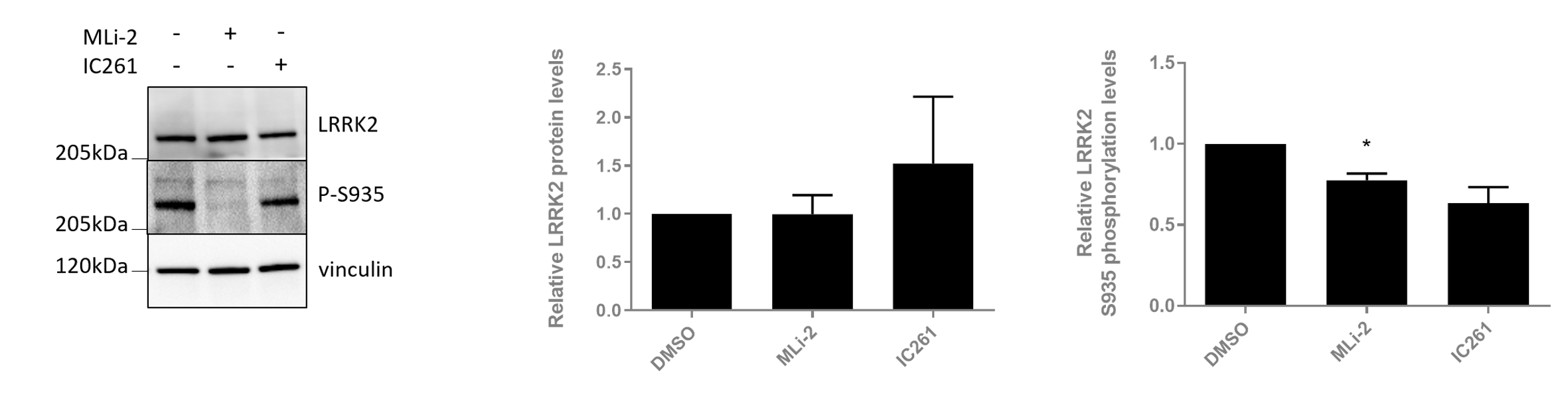

Supplement: Supplementary file 8 — High Resolution Image (TIF 4987 kb) [file 12035_2018_1449_MOESM4_ESM.tif]

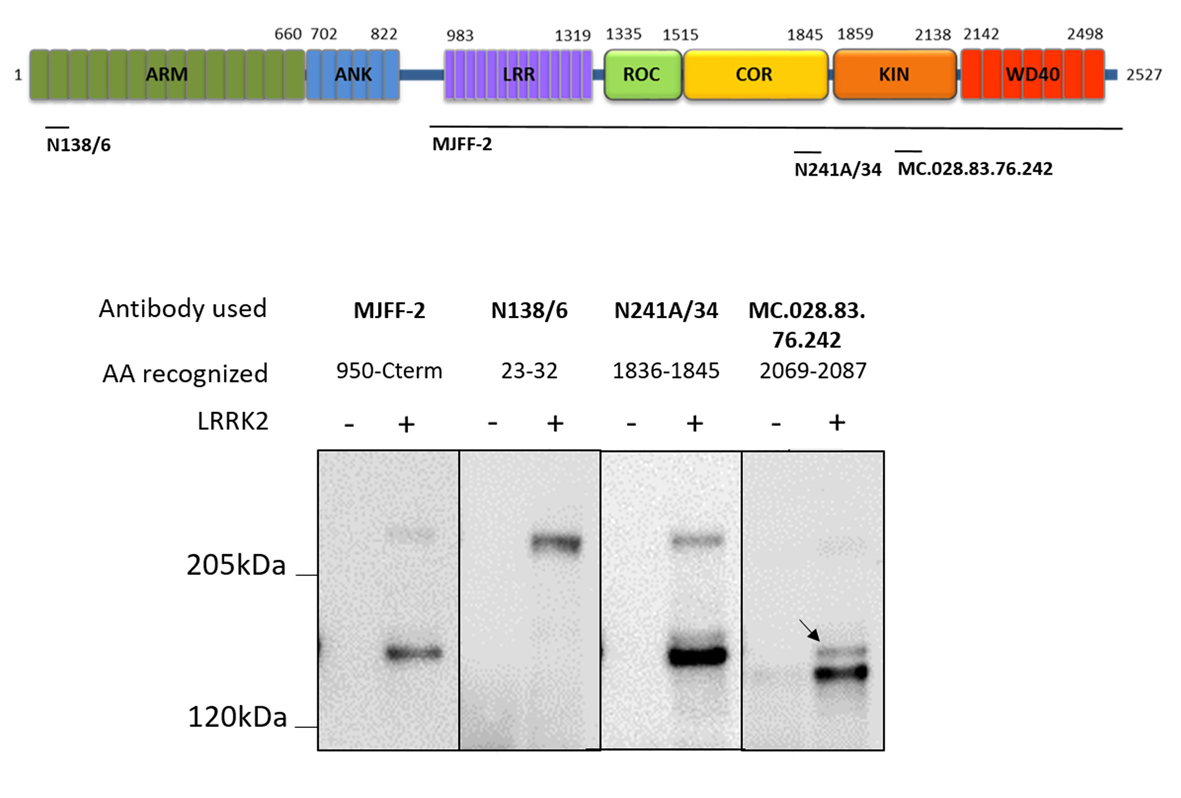

Supplement: Supplementary file 9 — The ~170kDa truncated LRRK2 variant in the kidney lacks an N-terminal part. Cell lysates from kidney tissue (C57Bl/6J WT or LRRK2-/- mice) were analyzed with immunoblotting using anti-LRRK2 MJFF-2 (ab133474), anti-LRRK2/Dardarin, N-terminus N138/6 (Neuromab 75-188), anti-LRRK2/Dardarin, Cterminus N241A/34 (Neuromab 75-253), anti-LRRK2 MC.028.83.76.242 (ab130277). The arrow indicates the presumed truncated LRRK2 variant (JPEG 257 kb) [file 12035_2018_1449_MOESM5_ESM.png]
